# Low incidence of atrial septal defects in nonmammalian vertebrates

**DOI:** 10.1111/ede.12322

**Published:** 2019-10-09

**Authors:** Bjarke Jensen, William Joyce, Martina Gregorovicova, David Sedmera, Tobias Wang, Vincent M Christoffels

**Affiliations:** ^1^ Department of Medical Biology, Amsterdam Cardiovascular Sciences University of Amsterdam Amsterdam The Netherlands; ^2^ Department of Bioscience, Zoophysiology Aarhus University Aarhus Denmark; ^3^ Institute of Anatomy, First Medical Faculty, Czech Academy of Sciences Charles University and Institute of Physiology Prague Czech Republic

**Keywords:** evolution, heart, patent foramen ovale, septation

## Abstract

The atrial septum enables efficient oxygen transport by separating the systemic and pulmonary venous blood returning to the heart. Only in placental mammals will the atrial septum form by the coming‐together of the *septum primum* and the *septum secundum*. In up to one of four placental mammals, this complex morphogenesis is incomplete and yields patent foramen ovale. The incidence of incomplete atrial septum is unknown for groups with the *septum primum* only, such as birds and reptiles. We found a low incidence of incomplete atrial septum in 11 species of bird (0% of specimens) and 13 species of reptiles (3% of specimens). In reptiles, there was a trabecular interface between the atrial septum and the atrial epicardium which was without a clear boundary between left and right atrial cavities. In developing reptiles (four squamates and one crocodylian), the *septum primum* initiated as a sheet that acquired perforations and the trabecular interface developed late. We conclude that atrial septation from the *septum primum* only results in a low incidence of incompleteness. In reptiles, the atrial septum and atrial wall develop a trabecular interface, but previous studies on atrial hemodynamics suggest this interface has a very limited capacity for shunting.

## Research Highlights

# We assessed the atrial septum in 11 species of birds and 13 species of reptiles.

# Birds and reptiles appear to have a lower incidence of incomplete atrial septum than placental mammals

## Graphical abstract

The atrial septum (arrow) of the African helmeted turtle. There is a low incidence of atrial septal defects in nonmammalian vertebrates.

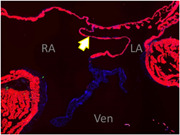

## 1 INTRODUCTION

The evolutionary success of terrestrial vertebrates relies on the ability to breathe air with lungs, and the evolution of a pulmonary circulation with a separate return of oxygenated pulmonary venous blood to the heart required some ability to separate the oxygen‐rich and oxygen‐poor blood within the heart (Johansen & Burggren, 1980; Perry & Sander, 2004). Separation of blood streams within the heart is governed by ridges and septa consisting of myocardial and connective tissues (Burggren, Farrell, & Lillywhite, 1998). Lungfishes (Dipnoi) have the atrial septum with the most characters that can be considered primitive, or evolutionarily old (Jensen, Wang, & Moorman, 2019). It is a meshwork of myocardial trabeculae anchored to a fold of connective tissue (Icardo et al., 2005; Klitgaard, 1978). Despite its flimsy appearance, it provides a surprisingly efficient separation of systemic and pulmonary venous blood streams (Burggren & Johansen, 1986; Icardo et al., 2005; Jensen et al., 2019; Klitgaard, 1978).

A complete atrial septum where there is no direct luminal continuity between the two atria appears to have evolved independently in anuran amphibians (frogs and toads) and in early amniotes, from the same myocardial and mesenchymal components as those in lungfishes (de Bakker, Wilkinson, & Jensen, 2015; Greil, 1908, 1913; Jensen et al., 2019; Robertson, 1913). Evolutionarily conserved transcription factors, such as *Nkx2*.*5*, *Tbx5*, and *Isl1*, confer identity to the myocardial and mesenchymal components (Jay, Degenhardt, & Anderson, 2016; Jensen et al., 2019; Khan & Jay, 2016; Steimle et al., 2018). In other amphibians (caecilians and salamanders), the state of development of the atrial septum varies substantially, but there is always a gap between the leading edge of the septum and the atrioventricular valves (de Bakker et al., 2015; Lewis & Hanken, 2017). Such morphology may be equated to an *ostium primum* defect if it was in a human. The atrial septum of placental mammals, however, is distinguished by the presence of the oval fossa, a circular depression on the right atrial face of the septum (Jensen et al., 2019; Röse, 1890; Rowlatt, 1990; Runciman, Gannon, & Baudinette, 1995). The rim of the oval fossa is the so‐called septum secundum, which develops late in gestation (R. H. Anderson, Spicer, Brown, & Mohun, 2014; S. Webb, Kanani, Anderson, Richardson, & Brown, 2001).

**Figure 1:**
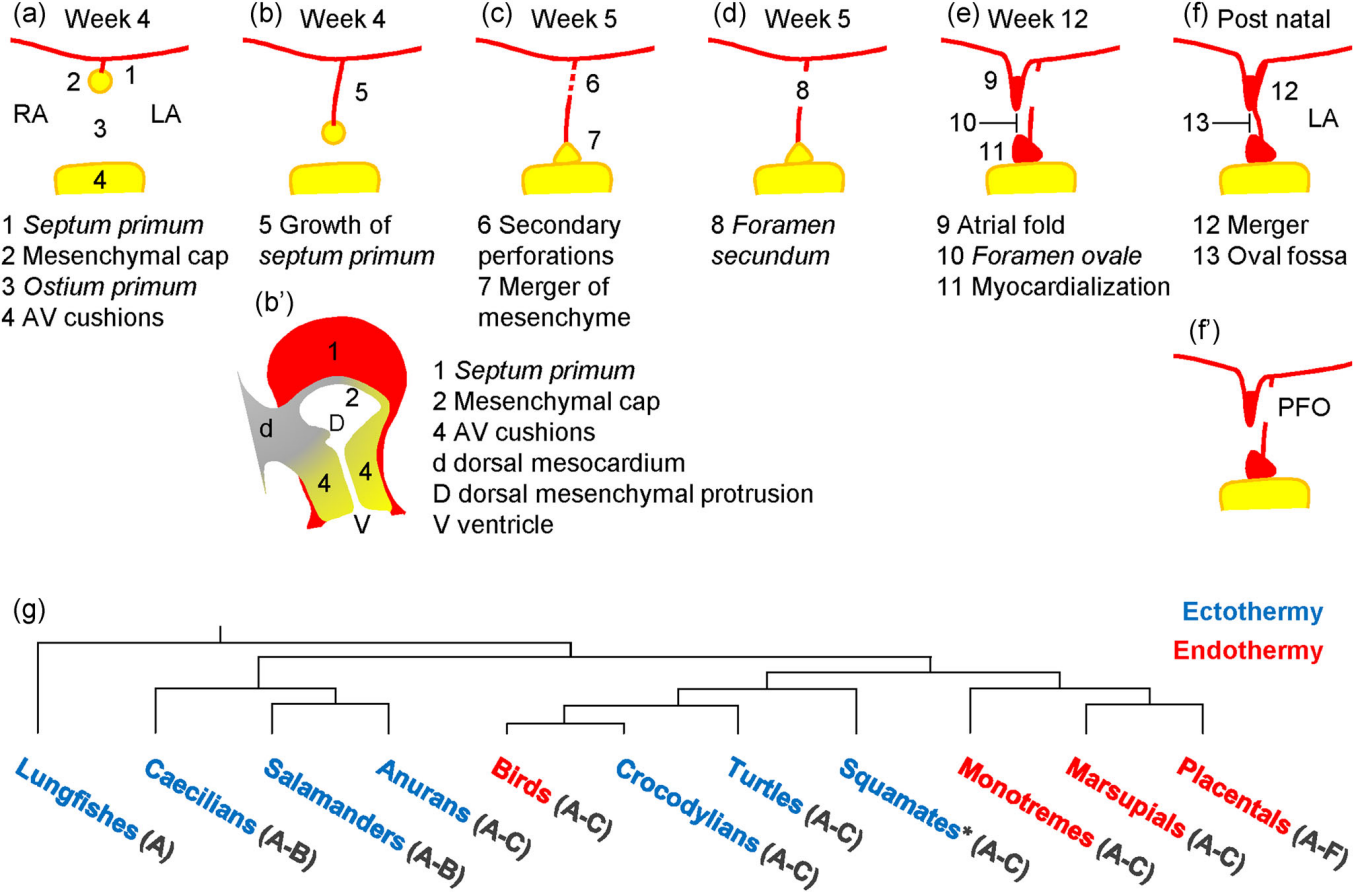
Atrial septum development (in human, a–f) and evolution (g). (a) By 4 weeks of human development, the *septum primum* (1) is a small thin sheet of myocardium with a cap of mesenchyme (2) and caudal to which is the *ostium primum* (3) and the atrioventricular cushions (4). (b) By growth of the atrial septum (5), the *ostium primum* becomes smaller. (b’) Sagittal view, showing the dorsal mesocardium (d) contributing the dorsal mesenchymal protrusion (D) to the closure of the *ostium primum*. (c) Secondary perforations (6) appear as the *ostium primum* is closed by the merger (7) of the mesenchyme that surrounds it (2, 4, D in B’). (d) The secondary perforations have coalesced to a single large *foramen secundum* (8). (e) By 12 weeks of gestation, a fold develops in the roof of the right atrium (9), which will give rise to the upper rim of the oval fossa of the formed heart. Cells of the mesenchymal cap have changed identity to myocardium and grown (11) and they will give rise to the lower rim of the oval fossa of the formed heart. Together these structures (9 and 11) form the rim of the *foramen ovale* (10). (f) After birth, relatively high pressures in the left atrium (LA) will press the remnants of the *septum primum* against upper part of the *septum secundum* (9) and this sets the stage for the merger of the *septum primum* and *septum secundum* (12). This closes the *foramen ovale* (10) which is now called the oval fossa (13). (f’) If the merger fails, partly or completely, there is patent foramen ovale (PFO). (g) Phylogenetic tree of major groups of tetrapod vertebrates and which indicates by the letters in the brackets which events and components of (a)–(f) occur in each group. Blue font indicates ectothermy and red font indicates endothermy. Note that events (d)–(f) only occur in placental mammals. Adapted from Jensen et al. (2019) [Color figure can be viewed at wileyonlinelibrary.com]

The gestational development of the atrial septum is most comprehensively studied in placental mammals, particularly in mouse and human (Wessels, 2016), but these events are highly similar between tetrapods (Jensen et al., 2019). Figure 1 schematizes the key events and components of human atrial septation. In the 3–4 weeks old human embryo, the atrial chambers “balloon” out from the primitive heart tube. At this stage, the atrial septum is only a ridge of mesenchyme in the center of the atrial roof (R. H. Anderson et al., 2014; Sylva, van den Hoff, & Moorman, 2014). Soon thereafter, the *septum primum* appears as a sheet of myocardium that carries the ridge of mesenchyme, now called the mesenchymal cap, on its leading edge (Figure 1a). In parallel, the atrial walls develop trabeculae which are strands of myocardium, only a few cells thick, that protrude into the chamber lumen. In adult mammals and birds, which are endothermic, these trabeculae will be relatively few and large, whereas in ectotherms the trabeculae are so numerous and small they form a spongy wall (Boukens et al., 2019). As the *septum primum* grows, it narrows the gap between its mesenchymal cap and the atrioventricular canal cushions (future valves; Figure 1b). This gap is called the *ostium primum*. It is closed when the mesenchymal cap and atrioventricular cushions merge, with additional mesenchyme being added from the so‐called dorsal mesenchymal protrusion (Figure 1c; Mommersteeg et al., 2006; Snarr et al., 2007; Wessels et al., 2000). When the closure fails, the lesion is called *ostium primum* defect and it occurs in less than 1 per mille of the human population (Hoffman & Kaplan, 2002). Before the closure of the *ostium primum*, around Week 5 of human gestation, perforations develop in the *septum primum*. These perforations enable oxygen‐rich blood to reach the cardiac left side (and subsequently the cranial and coronary circulation). The development of the atrial septum described thus far is evolutionarily conserved and applies to, besides placental mammals, to anuran amphibians, reptiles, birds, monotremes, and marsupials (Jensen et al., 2019).

In placental mammals, the perforations of the *septum primum* coalesce to a large hole, or a few such, referred to as the *foramen secundum* (Figure 1d; Franklin, Amoroso, Barclay, & Prichard, 1942; Macdonald, Carr, & Currie, 2007). The *foramen secundum* grows with the embryo and fetus, and in late development of human and sheep several hundreds of milliliters of blood flow across it every minute, or approximately a quarter of the cardiac output (D. F. Anderson et al., 1985; Kiserud, 2005). By Week 6 of human gestation, the mesenchyme that closed the *ostium primum*, grows, and changes identity to myocardium. This process leads to the formation of the lower, or inferior, rim of the future oval fossa (R. H. Anderson et al., 2014). Later, starting around Week 12 in human, the right atrial roof will fold in and develop a large crest of myocardium on the margin of the fold (Figure 1e; S. Webb et al., 2001). This process leads to the formation of the upper, or superior, rim of the future oval fossa, also known as the *crista dividens* (Kiserud, 2005). In human, the folding of the atrial roof is pronounced, whereas in mouse the upper rim is mostly a crest of myocardium (Jay, Degenhardt, & Anderson, 2016; Wessels, 2016). When the upper rim is less developed than the *foramen secundum*, there is an unhindered conduit for shunting across the atrial septum and such *septum secundum* defects occur in almost 1 *per mille* of the population (Hoffman & Kaplan, 2002). (In patent foramen ovale, the upper rim is well‐developed, only not fused with the *septum primum*). While nonplacental vertebrates do have secondary perforations in the *septum primum*, they do not develop the *foramen secundum* and the *septum secundum* required for *septum secundum* defects. In newly born placental mammals, left atrial pressure becomes substantially greater than right atrial pressure, and by this pressure gradient the *septum primum*, now commonly called the flap‐valve, is forced against the *septum secundum*. In most instances, the septa will come‐together in the course of weeks (Calvert, Rana, Kydd, & Shapiro, 2011; Nakanishi, Yoshiyama, & Homma, 2017). The attachment of the septa is likely by fibrosis promoted by mesenchyme derived from endocardium (Elliott, Gurtu, McCollum, Newman, & Wang, 2014). When the coming‐together is incomplete, there is so‐called patent foramen ovale. This occurs frequently in most placental mammals that have been investigated, in approximately one of four humans, rats, and mice (Calvert et al., 2011; Hagen, Scholz, & Edwards, 1984; LekanneDeprez et al., 1998; Nakanishi et al., 2017), one of five pigs (Hara et al., 2007), one of eight cows (Murakami, Hagio, & Nakai, 1990), and in just one of 36 harbor porpoises (Rowlatt & Gaskin, 1975).

Given the commonplace of patent foramen ovale in placental mammals, we wanted to know whether the incidence of incomplete atrial septation is also frequent when the atrial septum is formed from the *septum primum* only. To this end, we studied the incidence of incomplete atrial septum in birds (11 species), reptiles (13 species), and anuran amphibians (2 species), which have a complete atrial septum in contrast to caecilians and salamanders (Jensen et al., 2019).

## 2 MATERIALS AND METHODS

### 2.1 Specimens

We made use of previously published sections for hearts of adult birds (Kroneman et al., 2019), adult *Anolis carolinensis* (Jensen, Moorman, & Wang, 2014), adult *Python regius* (Jensen et al., 2017), and hearts of developing *Pantherophis guttatus* and *Norops sagrei* (B. Jensen et al., 2013) and *Varanus indicus* and *Varanus acanthurus* (Hanemaaijer et al., 2019). From (Joyce et al., 2019) we made use of sections of the following species: (*Pelomedusa subrufa* (*n* = 3; 20–35 g), *Chelodina mccordi* (*n* = 3; 14–15 g), *Pelodiscus sinensis* (*n* = 2; 5 g), *Cyclanorbis senegalensis* (*n* = 2; 0.2–0.45 kg), *Testudo hermanii* (*n* = 3; 25–27 g), *Chelonoidis carbonaria* (*n* = 3; 2.4–4.8 kg), *Chelydra serpentina* (*n* = 3; 30–35 g), and *Trachemys scripta* (*n* = 10; 0.3–1.7 kg), a skink, *Cyclodomorphus gerrardii*, (*n* = 1; 0.44 kg), an *Alligator mississippiensis* (*n* = 1; 2 kg), a spectacled caiman, *Caiman crocodilus* (*n* = 1; 4 kg), African clawed frogs (*Xenopus laevis*; *n* = 2; 50 g), and cane toads (*Rhinella marinus*; *n* = 2; 100–200 g). Fertilized surplus eggs of Siamese crocodile (*Crocodylus siamensis*) were obtained from The Crocodile Zoo Protivin between years 2013 and 2018 (5 embryos). Eggs were opened at weekly intervals. The incubation period of Siamese crocodile is around 68–80 days between temperature 28°C and 33°C. Eggs were incubated without rotation at 33 ± 1°C, which usually results in hatching at 68 days (Lang & Andrews, 1994). According to the Czech law, studies on embryos still contained in the eggs are exempt from approval of the Institutional Animal Care and Use Committee.

### 2.2 Histology

Fixation was by buffered 4% paraformaldehyde (pH 7.5) for 24 hr after which the hearts were stored in 70% ethanol. Subsequently, the hearts were imbedded in paraplast and sectioned in 10 µm thick sections. For each specimen, histological sections were collected at a constant distance. This distance was dependent on the size of the heart, such that the atria were present on at least 5 sections. For the smaller hearts, we typically collected one out of 20 sections, for the bigger hearts we typically collected one out of 100 (the actual collection rate is listed per specimen in Tables S1–S3). Sections were deparaffinized in baths of xylene and rehydrated in a series of alcohol from 100% to 50%. Most sections were stained either with picro‐sirius red (collagen red, myocardium orange) with 2 min differentiation in 0.01 M HCl or with hematoxylin–eosin with 10 min in hematoxylin followed by 10 min wash in running tap water, 1 min in 70% ethanol, 1 min in eosin, and 1 min differentiation in 96% ethanol. Sections of developing hearts were stained immunohistochemically for cardiac sarcomeric proteins. Briefly, cardiac muscle cTnI was detected with rabbit antibodies (Hytest, dilution between 1:250 and 1:600 dependent on the specimen, RRID:AB_154084) visualized by a fluorescently labeled secondary donkey–anti‐rabbit antibody (Thermo Fisher Scientific, dilution 1:200, RRID:AB_2535792), respectively, coupled to Alexa 488. Smooth muscle (SMA) was detected with a mouse antibody to smooth muscle actin (dilution 1:600, RRID:AB_476701; Sigma‐Aldrich) visualized by a fluorescently labeled secondary donkey anti‐mouse antibody coupled to Alexa 555 (dilution 1:250; RRID:AB_2536180; Thermo Fisher Scientific). Nuclei were stained with DAPI (dilution 1:1000; D9542; Sigma‐Aldrich). Sections of anole lizards were stained by *in situ* hybridization of digoxigenin‐labeled anti‐sense mRNA of *Anolis carolinensis Tnnt2* or *Myh7* and sections of American alligator for *Alligator mississippiensis cTnI* as described previously (Jensen et al., 2012; Jensen et al., 2017; Jensen et al., 2018; Moorman, Houweling, de Boer, & Christoffels, 2001). Briefly, we incubated sections overnight at +70°C with a probe concentration of 1 nmol/µl. The bound probe was detected immunologically using antidigoxigenin Fab fragment covalently coupled to alkaline phosphatase and NBT/BCIP was used as the chromogenic substrate (Roche). The immunohistochemistry on the Siamese crocodile was performed as in (Kvasilova, Gregorovicova, Kundrat, & Sedmera, 2019). Briefly, sections were incubated overnight at +4°C with mouse monoclonal IgM isotype to α sarcomeric actin (SA; 1:500; #A2172; Sigma). Following three rinses in phosphate buffered saline, we incubated the sections with horseradish peroxidase (HRP)‐conjugated goat anti‐mouse secondary antibody (1:200; Jackson ImmunoResearch #115‐035‐068). HRP activity was visualized using the diaminiobenzidine as substrate. Note that the images of the sections of the Siamese crocodile have been converted to gray scale and inverted such that stain appears light gray or white and such that signal resembles the signal that was detected with fluorescent microscopy.

### 2.3 Analysis

Besides the visual inspection by microscope for luminal continuities between the left atrium and the right atrium, we measured the length of the atrial septum in ImageJ (version 1.51a). We used the Segmented Line tool typically with some 20–40 segments to accommodate the curvatures of the septum. The length of the trabecular interface was measured as the straight line continuing from the end of the complete atrial septum to the epicardium of the atrial wall. To test whether there was a difference in the incidence of incomplete septation between mammals, birds, and reptiles, we performed *χ* ^2^ tests with Fisher's exact test (when frequency counts were less than 5) with values below 0.05 signifying significant difference. For human, we assumed the reported incidence of patent foramen ovale to be 263 persons per 965 persons with the patency having a median diameter of 5 mm (Hagen et al., 1984) in an atrial septum with an average width of 24 mm (Sweeney & Rosenquist, 1979). We, therefore, assumed that if 965 randomly chosen histological sections of the human atrial septum would be investigated, 54 sections ( = 263 sections × [5 mm/24 mm]) would reveal patency.

## 3 RESULTS

### 3.1 The atrial septum in birds

**Figure 2:**
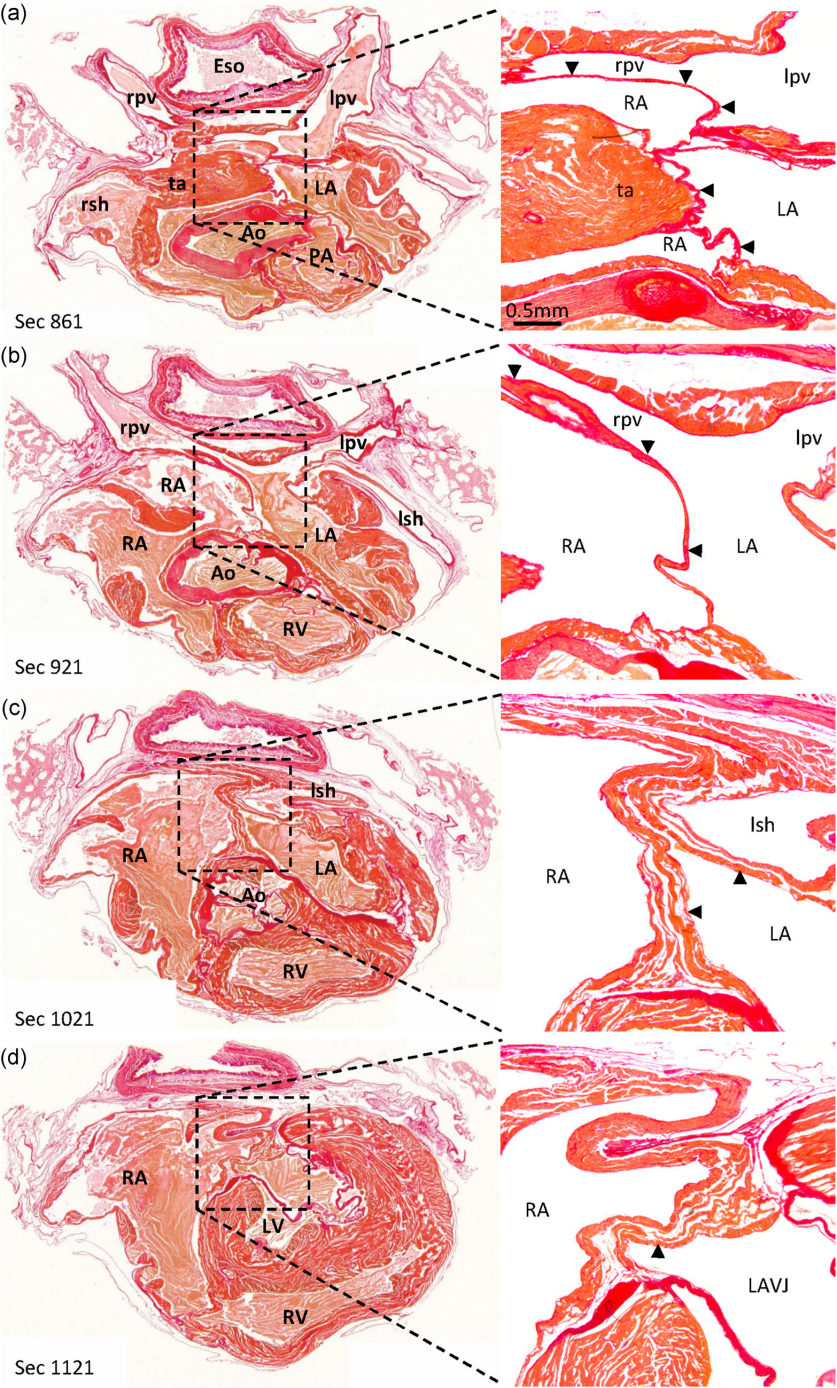
The atrial septum of the European green woodpecker (*Picus viridis*). Histological sections are 10 µm thick and stained with picro‐sirius red (collagen red, myocardium orange). Note that in the images on the right, blood has been painted over with white. (a) The atrial roof is dominated by the transverse arch (ta) attached to which is the thin atrial septum (arrowheads). The atrial septum is continuous with the myocardial wall between the right pulmonary vein (rpv) and the right atrium (RA). (b) There is no obvious border between the atrial septum and the myocardial wall of the right pulmonary vein. (c) Close to the ventricular base, the atrial septum thickens. The cavity of the left sinus horn (lsh) and left atrium (LA) are separated by a myocardial wall. (d) The myocardium of the atrial septum is continuous with myocardium of the atrioventricular junctions. Ao, aorta; LA, left atrium; LAVJ, left atrioventricular junction; lpv, left pulmonary vein; LV, left ventricle; PA, pulmonary artery; pv, pulmonary vein; rsh, right sinus horn; RV, right ventricle [Color figure can be viewed at wileyonlinelibrary.com]

Figure S1 documents that an analysis based on few equidistant sections is representative of an analysis based on all possible sections. Figure 2 shows histological sections at four different heights of the atrial cavities of one adult bird (the European green woodpecker; *Picus viridis*) and Table S1 gives an overview of the number of sections that were assessed in all bird species. In all species, the atrial septum was thin compared to the few large pectinate muscle in the atrial wall, and in particular compared to the transverse arch that dominates the roof of the atrial cavities (Figure 2a). An oval fossa was not observed on any section. From the sections of Figure 2, it can be seen that the atrial septum is continuous with the right pulmonary venous myocardium which separates blood of the pulmonary veins from the blood of the right atrium (Figure 2a,b). The atrial septum is also continuous with left atrial wall that partakes in the separation of the left atrial cavity from the left sinus horn, which is a conduit for the systemic venous blood (Figure 2c). The atrial septum is continuous with the myocardium of the atrioventricular junction (Figure 2d). In all of these settings, myocardial walls separate oxygen‐rich blood from oxygen‐poor blood and these walls were also analyzed for incompleteness, irrespective of whether the structure in question was the definitive atrial septum. Neither the atrial septum, nor any other associated structure, was incomplete or had a conduit for shunting on any section of any bird. The observed incidence of septal incompleteness (0/148 sections) was different (*p* = .001; Fisher's exact test) from the expected incidence for human (54/965 sections).

### 3.2 The atrial septum of reptiles

**Figure 3:**
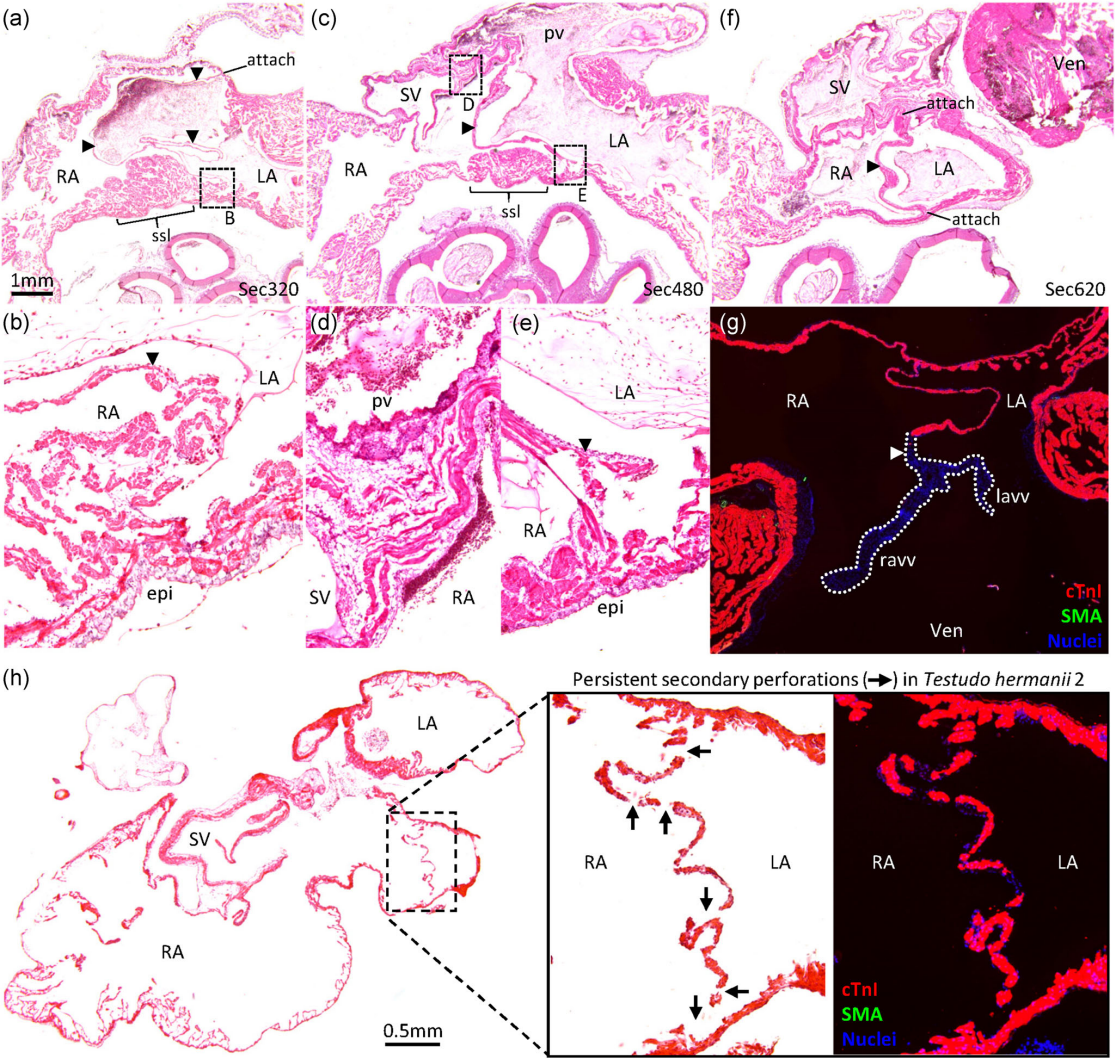
The atrial septum of turtles. Histological sections are 10 µm thick and stained with hematoxylin‐eosin. The species are *Cyclanorbis senegalensis* (a–f), *Pelomedusa subrufa* (g), and *Testudo hermanii* (h). The section plane in all images except (g) is approximately transverse and the images are oriented such that dorsal is up and the right is on the left. The section plane of (g) is frontal. (a,b) Cranial part of the highly trabecular atria. The atrial septum (arrowheads) is thin and a‐trabecular. In this section, it has a trabecular interface with the atrial wall ventrally (enlarged in (b)). There is a *septum secundum*‐like (ssl) large aggregate of trabeculae immediately to the right of the ventral attachment of the atrial septum to the atrial wall. (c–e) Near the opening of the sinus venosus (SV) to the right atrium (RA), the atrial septum has an a‐trabecular attachment dorsally (enlarged in (d)) and a trabecular interface ventrally (enlarged in (e)). (f) The atrial septum is complete in the atrioventricular canal and without any trabecular attachment. (g) The caudal‐most part of the atrial septum comprise connective tissue (white arrowhead) and anchors the left and right atrioventricular valve (lavv and ravv respectively). (h) The only section of this study on which we found (presumed) persistent secondary perforations (arrows). epi, epicardial side of the atrial wall; LA, left atrium; Ven, ventricle [Color figure can be viewed at wileyonlinelibrary.com]

**Figure 4:**
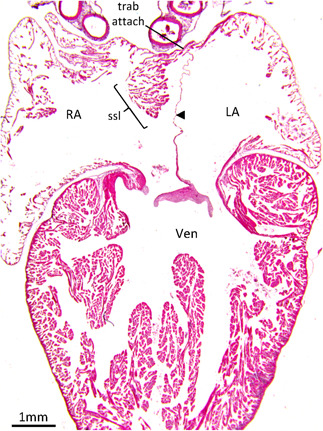
The atrial septum of the Pink‐tongued skink (*Cyclodomorphus gerrardii*). Histological section of 10 µm thickness in the frontal plane and stained with hematoxylin–eosin. The atrial septum (arrowhead) is thin, with a cranial trabecular attachment to the atrial wall (trab attach). The roof of the right atrial cavity is dominated by a *septum secundum*‐like large aggregate of trabeculae (ssl). LA, left atrium; RA, right atrium; Ven, ventricle [Color figure can be viewed at wileyonlinelibrary.com]

Figure 3a–f shows histological sections at three different heights of the atrial cavities of the Senegal flapshell turtle (*Cyclanorbis senegalensis*). The atria are very trabecular and this is also the case for all the reptile specimens of this study. The atrial septum appeared as a single sheet and it is approximately as thin as one of the surrounding trabeculae. It comprises myocardium, besides a thin endocardium (Figure 3g, heart of African helmeted turtle [*Pelomedusa subrufa*]). In some turtles, the atrial septum may have some smooth muscle (described in detail in Joyce et al. 2019). Only on one section (of the specimen Testudo 2 [*Testudo hermanni*]) did we find the atrial septum to have perforations that presumably were persistent secondary perforations and which were positioned cranially (Figure 3h). The observed incidence of septal incompleteness (1/157 sections) was different (*p* = .004; Fisher's exact test) from the expected incidence for human (54/965 sections). The attachment of the atrial septum to the atrial wall was by a trabecular interface on 76 of 129 sections in 26 of 29 specimens of reptiles (Table S2). This interface was found cranially, dorsally, and ventrally (Figure 3b,e). Erythrocytes were found between the trabeculae of the trabecular interface and the interface appeared without any clear boundary between the left and right atrial cavities. In the atrioventricular canal, there was no trabecular attachment (Figure 3f), the atrial septum was formed by connective tissue in the vicinity of the atrioventricular valve (Figure 3g; heart of African helmeted turtle [*Pelomedusa subrufa*]). In most specimens, we found a pronounced aggregate of trabeculae immediately to the right of the atrial septum, which resembled in position the *septum secundum*, or rim of the oval fossa, of the placental heart (Figure 3a,c; label “ssl”). This *septum secundum*–like structure was pronounced in turtles (Figure 3) and squamates (Figure 4). We did not observe any evidence of merging of the primary atrial septum with the *septum secundum*‐like structure. We did not observe the equivalent of the inferior rim of the oval fossa and an oval fossa was not found.

### 3.3 Late ontogenetic development of trabecular interface

**Figure 5:**
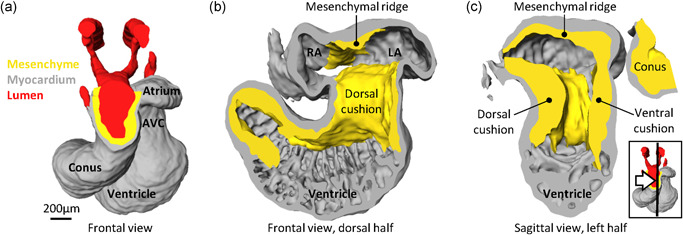
The earliest appearance of the atrial septum in the Corn snake (*Pantherophis*) two days after egg laying. (a) Frontal view of the heart where myocardium (gray), lumen (red) and mesenchyme (yellow) have been reconstructed. (b) Almost the same perspective as in A, only the lumen is not shown and the ventral half of the heart has been cut away. The roof of the atrium has a mesenchymal ridge. In later stages, the *septum primum* will grow from there and will carry on its leading edge the mesenchymal ridge as the mesenchymal cap. The future left atrium (LA) and right atrium (RA) are indicated. (c) The plane of this sagittal cut corresponds to the schematic drawing of Figure 1b’, only this heart is at an earlier stage (the insert shows the cut plane (black line) and perspective (white arrow)). The mesenchymal ridge and the dorsal and ventral cushion of the atrioventricular canal (AVC) constitute the perimeter of the *ostium primum*. The images are derived from a model that was published previously (Jensen et al., 2013) [Color figure can be viewed at wileyonlinelibrary.com]

**Figure 6:**
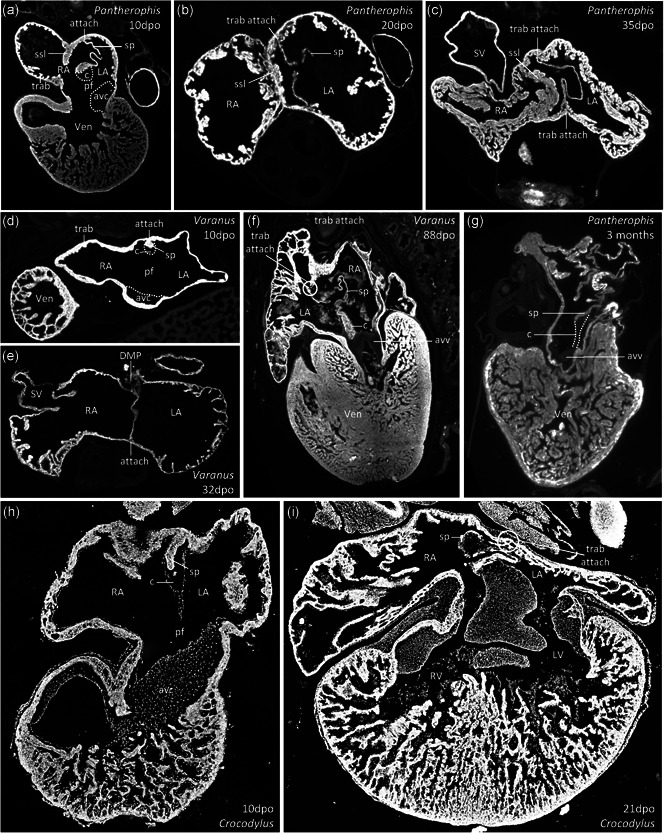
Development of the atrial septum in reptiles. Image (a) maybe compared to Figure 1 (b,c), and images (d) and (h) may be compared to Figure 1a. avc, atrioventricular cushion; avv, atrioventricular valve; c, connective tissue; DMP, dorsal mesenchymal protrusion; LA, left atrium; pf, primary foramen; RA, right atrium; sp, *septum primum*; ssl, *septum secundum*‐like structure; SV, sinus venosus; trab, trabeculae; Ven, ventricle. (The sections of (a)‐(g) were visualized with fluorescent microscopy, whereas the sections of (h)‐(i) were visualized with bright‐field microscopy. Images (h)‐(i) were then converted to gray scale and inverted to appear more similar to images (a)‐(g))

We investigated the development of the atrial septum to better understand how and when its trabecular interface to the atrial wall develops. Development of the atrial septum was studied in the Corn snake (*Pantherophis guttatus*; Table 1; Figure 5, 6a–c, g), the Brown anole (*Norops sagrei*; Table 2), in the Mangrove monitor (*Varanus indicus*; Table 3, Figure 6d), the Spiny‐tail monitor (*Varanus acanthurus*), and in the Siamese crocodile (*Crocodylus siamensis*; Table 4, Figure 6h–i; of the two species of monitors, we focused on the data from the Mangrove monitors as that series had more specimens across a broader period of development than the series of the Spiny‐tail monitor). Tables 1, 2, 3, 4 give an overview of developmental changes to components of the atrial septum. Atrial septation was similar in all five species. The earliest appearance of the atrial septum was a ridge of mesenchyme spanning the atrial roof that was continuous with the dorsal and ventral cushion of the atrioventricular canal (this early stage was only seen in the Corn snake and the Mangrove monitor; Figure 5). Shortly thereafter, the myocardial *septum primum* started to develop from underneath the mesenchymal ridge. By growth of the septum, the mesenchymal ridge was carried forward on the crest of the septum as the so‐called mesenchymal cap (all species; Figure 6a, d, h). Concurrent with the initial growth of the *septum primum*, trabeculae in the atrial chambers started to appear (trabeculae in the ventricle were already well established at this stage). The atrial septum had the appearance of a single sheet and its junction with the atrial wall was without trabeculae (Figure 6a,d, h).

**Table 1:** Ontogeny of atrial septal components in *Pantherophis guttatus*

Days post oviposition	2	10	12	14	16	20	26	35	42
Septum primum		+	+	+	+	+	+	+	+
Mesenchymal cap	ridge	+	+	merged	merged	merged	merged	merged	merged
Primary foramen		+	+	closed	closed	closed	closed	closed	closed
Perforations		+	+	+	+	+	+	+	+
Atrial trabeculation	–	+	+	+	+	+	+	+	+
Trabecular interface		no	no	yes	yes	yes	yes	yes	yes

**Table 2:** Ontogeny of atrial septal components in *Norops sagrei*

Sanger stage	5	7	9	12	17	19
Septum primum	+	+	+	+	+	+
Mesenchymal cap	+	+	merged	merged	merged	merged
Primary foramen	+	+	closed	closed	closed	closed
Perforations	–	–	+	+	+	+
Atrial trabeculation	–	–	+	+	+	+
Trabecular interface	no	no	no	no	no	no/yes

**Table 3:** Ontogeny of atrial septal components in *Varanus indicus*

Days post oviposition	10	24	32	56	73	88	144
Septum primum	+	+	+	+	+	+	+
Mesenchymal cap	+	some myo	some myo	some myo	myo	myo	myo
Primary foramen	+	+	closed	closed	closed	closed	closed
Perforations	–	+	+	+	+	+	+
Atrial trabeculation	+	+	+	+	+	+	+
Trabecular interface	no	no	no	yes	yes	yes	yes

**Table 4:** Ontogeny of atrial septal components in *Crocodylus siamensis*

Days post oviposition	10	21	30	42	54
Septum primum	+	+	+	+	+
Mesenchymal cap	+	some myo	some myo	merged	merged
Primary foramen	+	+	closed	closed	closed
Perforations	−	+	+	+	‐?
Atrial trabeculation	+	+	+	+	+
Trabecular interface	no	yes	yes	yes	yes

Growth of the atrial septum moved the mesenchymal cap in proximity of the atrioventricular cushions (Figure 6a). Concomitantly, perforations started to develop in the cranial part of the *septum primum* (Figure 6a,b). Shortly hereafter, the mesenchymal cap made contact with the atrioventricular cushions and merged with them and thereby closed the *foramen primum* (Figure 6i). In later stages, the mesenchymal cap would become less distinct, but remained histologically recognizable as a nonmyocardial sheet in the same plane as the myocardial atrial septum and in between the myocardial atrial septum and the atrioventricular valves (Figure 6g; label “c”). In both species of monitors and the Siamese crocodile, the mesenchymal cap acquired a mostly myocardial phenotype, starting from 24 days past oviposition (dpo) in the Mangrove monitor. It remained recognizable as a particularly thick part of the atrial septum immediately cranial to the atrioventricular valve (Figure 6f, compare to Figure 6g). The monitors also had a large dorsal mesenchymal protrusion, which acquired substantial amounts of myocardium, starting from 24 dpo in the Mangrove monitor. It contributed to a broad attachment of the atrial septum to the dorsal wall of the atria (Figure 6e).

The large rim in the atrial roof that we presume to be a precursor structure of the *septum secundum*, was observed in all species of the noncrocodilian reptiles and was most pronounced in the Corn snake (Figure 6a–c). In sections that show both the *septum primum* and the precursor *septum secundum*, the precursor *septum secundum* appeared much more developed (Figure 6a–c). However, only the *septum primum* made contact with the atrioventricular cushions, whereas the precursor *septum secundum* tapered off immediately to the right of the atrioventricular canal (Figure 6a). In the Siamese crocodile, the precursor structure of the *septum secundum* was poorly developed.

The atrial wall became progressively more trabeculated in gestation in all five species (Figure 6). This was the least pronounced in the brown anole, the hatchlings of which are the smallest of the five species. In one out of three hatchlings of the brown anole, we observed a few sections where there were a few trabeculae ventrally between the atrial septum and the atrial wall. Such trabecular interface was more pronounced and common in the Corn snake, monitors, and the Siamese crocodile and was observed ventrally, cranially, and dorsally (Figure 6b,c,f,i; Tables 1, 2, 3, 4).

### 3.4 The atrial septum in anuran amphibians

**Figure 7:**
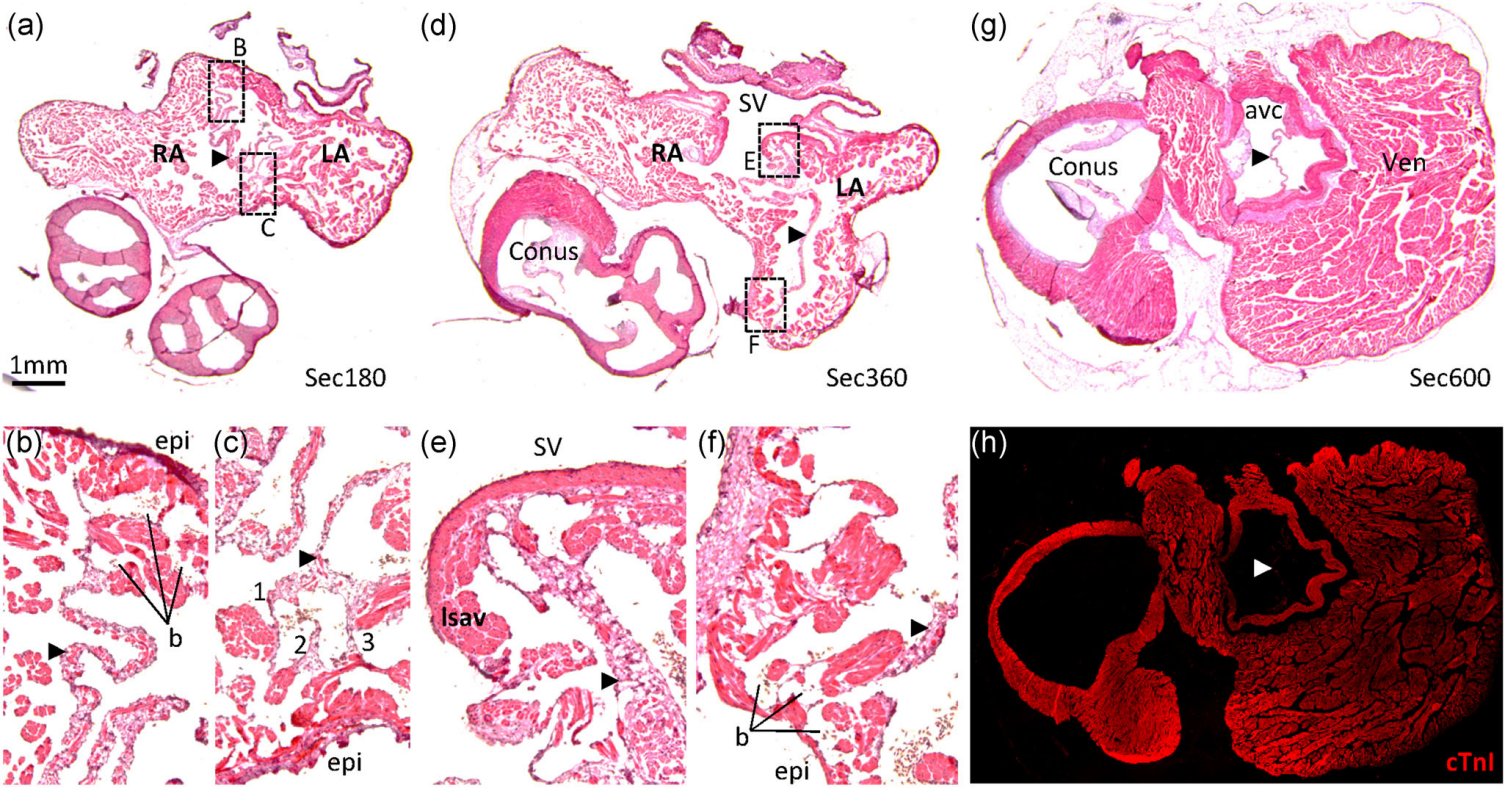
The atrial septum of the Cane toad (*Rhinella marina*). Histological sections are 10 µm thick and stained with hematoxylin‐eosin. (a–c) Cranial part of the highly trabecular atria. The atrial septum (arrowhead) is as thick, if not thicker, than the surrounding trabeculae and has a high content of connective tissue. It has a trabecular attachment to the atrial wall dorsally (b) and ventrally (c). In image (b), notice the blood (b) between trabeculae and the multiple branches (1,2,3) of the atrial septum (c). (d–f) Near the opening of the sinus venosus (SV) to the right atrium (RA), the atrial septum attaches to the left leaflet of the sinuatrial valve (lsav) (e) and has a trabecular attachment to the atrial wall ventrally (f). (g,h) The atrial septum is complete in the atrioventricular canal (g) where it is without cardiac troponin I (white arrowhead, h) and is therefore considered to be without myocardium. (g and h is the same section, which was first stained immunohistochemically and imaged (h) and only thereafter was it stained with hematoxylin‐eosin (g)). The specificity of the cTnI detection is revealed, for instance, by the lack of stain of the conus and avc valves that can be seen in g. avc, atrioventricular canal; epi, epicardial side of the atrial wall; LA, left atrium; Ven, ventricle [Color figure can be viewed at wileyonlinelibrary.com]

Figure 7 shows histological sections at three different heights of the atrial cavities of the cane toad (*Rhinella marina*). In all amphibians that we investigated, the atria were very trabecular. The atrial septum was in some parts marginally thicker than the surrounding trabeculae. It comprised myocardium and connective tissue. On sections, the myocardium of the septum appeared as multiple rounded or oval islands, much like the surrounding trabeculae, rather than a continuous sheet. An oval fossa was not observed on any section. On some sections, the attachment of the atrial septum to the atrial wall had a trabecular interface dorsally (Figure 7b), ventrally (Figure 7c,f), and cranially (not shown). A trabecular interface was found on 21 of 28 sections in 5 of 5 specimens of amphibians (Table S3). Erythrocytes were found between the trabeculae of the trabecular interface and the interface appeared without any clear boundary between the left and right atrial cavities. On many sections, it appeared as if the atrial septum branched into the trabecular atrial wall (exemplified in Figure 7c). In the atrioventricular canal, there was no trabecular interface between the atrial septum and the epicardium. There, the atrial septum was formed by connective tissue (presumably derived from mesenchymal cap tissue) in the vicinity of the atrioventricular valve (Figure 7g,h).

## 4 DISCUSSION

Birds and reptiles form the atrial septum from the *septum primum* only and our findings suggest that their atrial septum may have a lower incidence of incompleteness than the incidence of patent foramen ovale of most placental mammals (human, mouse, rat, pig, cow). In the reptiles and anuran amphibians, almost every specimen had a trabecular interface between the atrial septum and the atrial wall. Our developmental investigations suggest that the trabecular interface results from the extensive trabeculation of the atrial wall. At least to us, the presence of the trabecular interface should not be equated to an incompleteness of the atrial septum.

### 4.1 Incidence of atrial septal defects in nonmammals

Overt septal defects are not common in the reptiles and anuran amphibians. Only in one specimen of tortoise, did we find a section with a few perforations that probably persisted since gestation (they were found in the dorso‐cranial part of the septum where the secondary perforations develop). The secondary perforations are normally closed by growth of surrounding myocardial, endothelial, and mesenchymal tissues (Hendrix & Morse, 1977; Jensen et al., 2019; Sedmera et al., 2006).

We never observed the equivalent of an *ostium primum* defect, that is a defect which is enclosed by mesenchymal tissues and found immediately cranial to the atrioventricular valves. We propose atrial septal defects are not common in reptiles, although one case has been described in the Komodo dragon where a large hole was found in the muscular part of the atrial septum (Pizzi et al., 2009). In birds, the congenital cardiac malformations that can be recognized by gross morphology are rare and are thought to occur at approximately at the same rate as in human (Taussig, 1988; Tudor, 1979). It is not clear to what extent persistent secondary foramens can be detected by gross morphological inspection. We propose that persistent secondary perforations, such as the ones we found in a turtle, are not likely to be frequent in birds.

Patent foramen ovale is typically assessed by gross morphological inspection in relatively large hearts (human, pig, cow). In contrast, we relied exclusively on histology of small to medium‐sized hearts. Such differences may have impacted the detection of incomplete parts of the atrial septum. Previously, G. J. W. Webb (1979) studied, by gross morphology, large hearts of eight crocodiles, and found in the atria of the three largest specimens a few “openings” of approximately 1 mm in diameter through which the left and right atrial cavities could potentially exchange blood. This could suggest a relatively high incidence of persistent secondary foramens in crocodiles. However, G. J. W. Webb (1979) further reported that the “openings are continuous with trabecular spaces of the left atrium” so it is not clear whether the “openings” are part of the atrial septum, or part of what we describe as the trabecular interface between the atrial septum and the atrial wall. In contrast to gross anatomical dissections, where findings can be ambiguous, studies in small mammals demonstrate that histology is efficient in identifying secondary perforations and the foramen ovale (Briggs, Kakarla, & Wessels, 2012; Elliott et al., 2014; Runciman et al., 1995). We, therefore, consider it unlikely that there has been a major impact of the manner of investigation and specimen size on the incidences of atrial septal incompleteness we report here.

### 4.2 Evolution of the full atrial septum of amniotes and anuran amphibians

The atrial septum of the reptiles and anuran amphibians is always a thin sheet comprised of mostly myocardium (de Graaf, 1957; Jensen et al., 2019; Sedmera et al., 2003). In the amphibians, the appearance on sections of the septal myocardium as multiple rounded or oval islands of myocardium, suggests that the septum is formed as an extensive trabecular meshwork. The gaps of the meshwork are then closed later by growth, with a potentially great role of the endocardium. In salamanders, the atrial septum also has the appearance of a myocardial meshwork (Davies & Francis, 1941; Lewis & Hanken, 2017). In the amniotes, the myocardium of the atrial septum appears as a single sheet which acquires few perforations relative to the amphibians. These differences between amniotes and anuran amphibians are consistent with the view that the two groups evolved full atrial septation independently (Jensen et al., 2019; Lewis & Hanken, 2017).

### 4.3 A trabecular interface is common in ectotherms only

The trabecular interface between the atrial septum and the epicardial surface of the atrial wall was found in almost all specimens of amphibians and reptiles. Erythrocytes were found in the spaces between the trabeculae of the trabecular interface. The trabecular interface is therefore a likely conduit for shunting between the left and right atrium. However, the capacity for shunting could be small; in *in situ* perfused hearts of Burmese python and Savannah monitors, which have a functionally divided ventricle, elevation of the left atrial pressure does not increase flow in the pulmonary artery (no left‐to‐right shunting) and elevation of right atrial pressure does not increase flow in the systemic arteries (no right‐to‐left shunting; Joyce, Axelsson, Altimiras, & Wang, 2016; Wang, Altimiras, & Axelsson, 2002). In the yellow anaconda (*Eunectes notaeus*), elevation of left or right atrial pressure does not impact on the pressure of the other atrium, confirming that the snake atria exhibit very limited shunting, if any at all (Joyce, Axelsson, & Wang, 2017). It is remarkable that birds appear to be without the trabecular interface and only develop a few and very large trabeculae in the atrial wall. The atrial wall architecture of birds (and mammals) is more compact than in ectothermic vertebrates (Boukens et al., 2019) and this may limit the formation of a trabecular interface between the atrial septum and the atrial wall.

### 4.4 Evolutionary precursors of the *septum secundum*

We have previously shown in a few species of reptiles, that the right atrium has an aggregate of trabecular myocardium at the position that corresponds to the position of the superior rim of the *septum secundum* in placental mammals (Jensen et al., 2019). We show here, that this aggregate is a common feature of noncrocodylian reptile hearts. In the amphibians, there was no obvious *septum secundum*‐like structure. In some of the reptiles investigated here, for example the skink (*Cyclodomorphus gerrardii*; Figure 4), this *septum secundum*‐like structure is exceptionally pronounced and its size relative to the surrounding atrial wall corresponds well to the superior rim of the *septum secundum*. The *septum secundum* associates with a localized folding in of the atrial roof which is more pronounced in human than in mouse (R. H. Anderson et al., 2014). A similar folding of the atrial roof was not observed in the reptiles. Instead, a broad medial part of the atrial roof is low compared to the lateral parts of the atria, and this “gully” holds the major arteries.

In mammals, the dorsal mesenchymal protrusion adds mesenchyme to the closure of the *ostium primum* and most of which will change identity to myocardium (Briggs et al., 2012). We have previously shown that the dorsal mesenchymal protrusion of anole lizards has some capacity to change identity to myocardium (Jensen et al., 2019). We show here, that this capacity appears particularly pronounced in the monitors. Monitors, in contrast to other lizards, develop so large ventricular septa that the ventricle is functional separated into a high‐pressure left ventricle and a low‐pressure right ventricle and thus much like the ventricles of mammals and birds (Burggren & Johansen, 1982; Jensen et al., 2014; Joyce et al., 2016). Extensive myocardialization of mesenchymal tissues is also characteristic of mammals and birds (van den Hoff, Kruithof, Moorman, Markwald, & Wessels, 2001).

We consider it likely that the heart of reptiles has structures that are homologous to structures of the placental mammal heart that contribute to the *septum secundum*. Nonetheless, a structure akin to the oval fossa is not readily observed in reptiles. This reflects the lesser development of the lower rim of the oval fossa and lack of coming‐together of the *septum secundum*‐like structure and the *septum primum*, in addition to the highly trabecular wall architecture in the reptiles.

## CONFLICT OF INTEREST STATEMENT

The authors declare there are no conflict of interests.

## AUTHOR CONTRIBUTIONS

Data acquisition by B. J., W. J., and M. G.; analyses and writing of manuscript by B. J., W. J., D. S., T. W., and V. M. C.

## Supporting information

Supporting information

Supporting information
