# Supplementary material for: Low incidence of atrial septal defects in nonmammalian vertebrates
Source: Evol Dev. 2019 Oct 9;22(3):e12322. doi: 10.1111/ede.12322 (PMC9285691; doi:10.1111/ede.12322)
Supplement: Supplementary file 1 — Supporting information [file EDE-22-241-s002.docx]

Supplementary Tables

Supplementary Table 1. Assessed sections in birds. The category Sampling is the number of collected sections (n/) relative to the number of sections that were cut (/n).

| Species | Intact | Incomplete | Incidence | Damaged | Sampling |
| --- | --- | --- | --- | --- | --- |
| Barn swallow | 5 | 0 | 0 | 0 | 1/30 |
| Budgerigar | 16 | 0 | 0 | 0 | 1/25 |
| Mallard | 17 | 0 | 0 | 2 | 1/30 |
| Collared dove | 12 | 0 | 0 | 0 | 1/60 |
| Common kestrel | 18 | 0 | 0 | 5 | 1/20 |
| Euroasian coot | 19 | 0 | 0 | 0 | 1/20 |
| Green woodpecker | 20 | 0 | 0 | 0 | 1/20 |
| Lesser redpoll | 11 | 0 | 0 | 0 | 1/20 |
| Swift | 13 | 0 | 0 | 1 | 1/20 |
| Blackbird | 7 | 0 | 0 | 6 | 1/30 |
| Western jackdaw | 10 | 0 | 0 | 1 | 1/10 |

Supplementary Table 2. Analyzed sections in hatched reptiles. Values for AS, Trab, and Trab/AS are averages (standard deviations are given in supplementary table 1). The category Incomplete includes septal defects and the trabecular interface. The category Sampling is the number of collected sections (n/) relative to the number of sections that were cut (/n).

| Specimen | Intact | Incomplete | Incidence | AS (mm) | Trab (mm) | Trab/AS (%) | Damaged | Sampling |
| --- | --- | --- | --- | --- | --- | --- | --- | --- |
| *Cyclodomorphus* | 2 | 2 | 1 | 2.71 | 0.09 | 4.46 | 0 | 1/60 |
| *Python* | 6 | 5 | 0.83 | 3.47 | 0.26 | 7.33 | 0 | 1/50 |
| *Pantherophis* | 11 | 8 | 0.73 |  |  | 11.20 | 0 | 1/10 |
| *Chelydra* 1 | 4 | 1 | 0.25 | 6.86 | 1.90 | 27.76 | 1 | 1/120 |
| *Chelydra* 2 | 4 | 2 | 0.50 | 12.61 | 0.32 | 2.69 | 0 | 1/80 |
| *Chelydra* 3 | 3 | 1 | 0.33 | 7.58 | 0.27 | 3.55 | 1 | 1/120 |
| *Chelodina* 1 | 5 | 3 | 0.60 | 2.24 | 0.12 | 5.37 | 1 | 1/20 |
| *Chelodina* 2 |  |  |  |  |  |  | 5 | 1/40 |
| *Chelodina* 3 | 2 | 2 | 1 | 1.88 | 0.20 | 10.99 | 1 | 1/60 |
| *Cyclanorbis* 1 | 2 | 1 | 0.50 | 4.82 | 0.19 | 4.0 | 3 | 1/100 |
| *Cyclanorbis* 2 | 3 | 2 | 0.67 | 10.61 | 0.42 | 4.28 | 0 | 1/160 |
| *Pelomedusa* 1 | 3 | 2 | 0.67 | 4.05 | 0.10 | 6.45 | 0 | 1/40 |
| *Pelomedusa* 2 | 3 | 0 | 0 |  |  |  | 2 | 1/50 |
| *Pelomedusa* 3 | 4 | 1 | 0.25 | 0.63 | 0.08 | 13.19 | 0 | 1/60 |
| *Testudo* 1 | 3 | 1 | 0.33 | 3.20 | 0.34 | 10.52 | 0 | 1/50 |
| *Testudo* 2 | 5 | 1 | 0.20 | 4.47 | 0.09 | 2.08 | 0 | 1/40 |
| *Testudo* 3 | 7 | 1 | 0.14 | 2.62 | 0.19 | 7.35 | 0 | 1/30 |
| *Chelonoidis* 1 | 6 | 5 | 0.83 | 7.27 | 2.98 | 44.19 | 1 | 1/80 |
| *Chelonoidis* 2 | 6 | 3 | 0.50 | 9.23 | 1.72 | 20.45 | 3 | 1/120 |
| *Chelonoidis* 3 | 5 | 4 | 0.80 | 13.75 | 1.74 | 13.96 | 1 | 1/80 |
| *Trachemys* 1 | 3 | 2 | 0.67 | 6.27 | 0.95 | 16.82 | 1 | 1/4 |
| *Trachemys* 2 | 8 | 8 | 1 | 9.58 | 1.08 | 8.54 | 0 | 1/80 |
| *Trachemys* 3 | 4 | 3 | 0.75 | 6.86 | 0.72 | 18.90 | 3 | 1/80 |
| *Trachemys* 4 | 4 | 2 | 0.50 | 3.85 | 0.38 | 11.22 | 0 | 1/80 |
| *Trachemys* 5 | 5 | 4 | 0.80 | 11.15 | 0.42 | 3.74 | 0 | 1/100 |
| *Trachemys* 6 | 3 | 1 | 0.33 | 10.64 | 0.15 | 1.44 | 3 | 1/80 |
| *Trachemys* 7 |  |  |  |  |  |  | 1 | NA |
| *Trachemys* 8 | 2 | 0 | 0 |  |  |  | 0 | 1/? |
| *Trachemys* 9 | 7 | 4 | 0.57 | 6.36 | 0.40 | 6.33 | 6 | 1/80 |
| *Trachemys* 10 | 8 | 6 | 0.75 | 4.01 | 0.69 | 18.08 | 0 | 1/60 |
| *Caiman* 1 | 2 | 0 | 0 |  |  |  | 1 | 1/5 |

Supplementary Table 3. Assessed sections in Anuran amphibians. The category Incomplete includes septal defects and the trabecular interface. The category Sampling is the number of collected sections (n/) relative to the number of sections that were cut (/n).

| Specimen | Intact | Incomplete | Incidence | AS (mm) | Trab (mm) | Trab/AS (%) | Damaged | Sampling |
| --- | --- | --- | --- | --- | --- | --- | --- | --- |
| *Rhinella* 1 | 2 | 2 | 1 | 8.72 | 0.61 | 6.9 | 4 | 1/80 |
| *Rhinella* 2 | 10 | 7 | 0.70 | 8.14 | 0.62 | 8.5 | 2 | 1/60 |
| *Rhinella* 3 | 11 | 9 | 0.82 | 6.17 | 0.44 | 7.8 | 1 | 1/60 |
| *Xenopus* 1 | 2 | 1 | 0.50 | 4.42 | 0.21 | 4.8 | 1 | 1/100 |
| *Xenopus* 2 | 3 | 2 | 0.67 | 2.69 | 0.25 | 12.7 | 0 | 1/100 |
