# Supplementary figures and images for: Low incidence of atrial septal defects in nonmammalian vertebrates
Source: Evol Dev. 2019 Oct 9;22(3):e12322. doi: 10.1111/ede.12322 (PMC9285691; doi:10.1111/ede.12322)

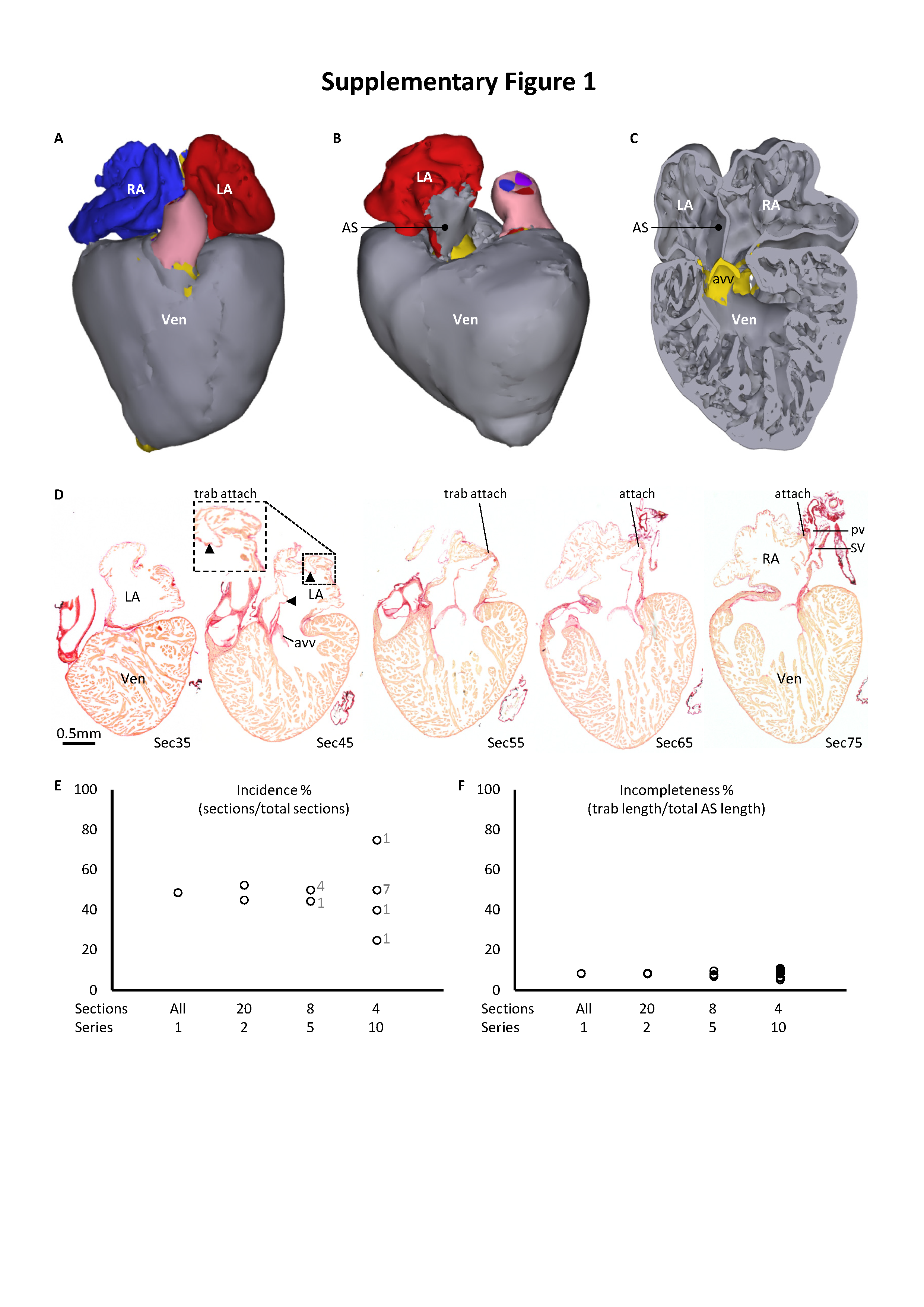

Supplement: Supplementary file 2 — Supporting information [file EDE-22-241-s001.tiff]
